# A Fuzzy Cooperative Localisation Framework for Underwater Robotic Swarms

**DOI:** 10.3390/s20195496

**Published:** 2020-09-25

**Authors:** Adham Sabra, Wai-Keung Fung

**Affiliations:** 1 School of Engineering, Robert Gordon University, Aberdeen AB10 7GJ, UK; a.a.k.sabra@rgu.ac.uk; 2 Cardiff School of Technologies, Cardiff Metropolitan University, Llandaff Campus, Cardiff CF5 2YB, UK

**Keywords:** underwater wireless sensor networks, underwater swarm robotics, autonomous underwater vehicles, underwater localisation, cooperative navigation, fuzzy systems

## Abstract

This article proposes a holistic localisation framework for underwater robotic swarms to dynamically fuse multiple position estimates of an autonomous underwater vehicle while using fuzzy decision support system. A number of underwater localisation methods have been proposed in the literature for wireless sensor networks. The proposed navigation framework harnesses the established localisation methods in order to provide navigation aids in the absence of acoustic exteroceptive sensors navigation aid (i.e., ultra-short base line) and it can be extended to accommodate newly developed localisation methods by expanding the fuzzy rule base. Simplicity, flexibility, and scalability are the main three advantages that are inherent in the proposed localisation framework when compared to other traditional and commonly adopted underwater localisation methods, such as the Extended Kalman Filter. A physics-based simulation platform that considers environment’s hydrodynamics, industrial grade inertial measurement unit, and underwater acoustic communications characteristics is implemented in order to validate the proposed localisation framework on a swarm size of 150 autonomous underwater vehicles. The proposed fuzzy-based localisation algorithm improves the entire swarm mean localisation error and standard deviation by 16.53% and 35.17%, respectively, when compared to the Extended Kalman Filter based localisation with round-robin scheduling.

## 1. Introduction

**Figure 1:**
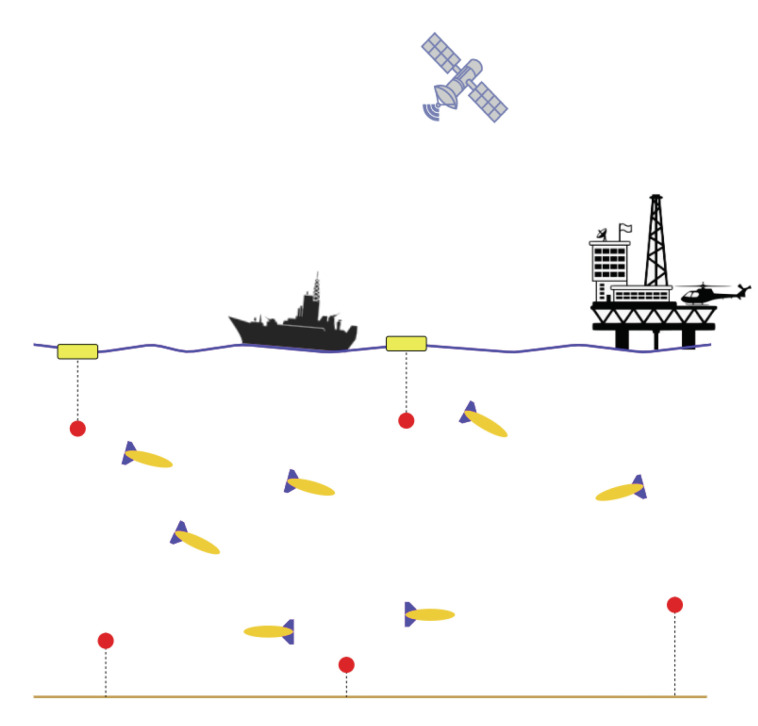
Underwater wireless network of Autonomous Underwater Vehicles (yellow vessels with blue fins) and anchored sensors (red).

Over the past two decades, swarm robotics have been widely investigated and robotic swarms have been proven to be more efficient in solving complicated tasks or tasks that require wide spatial coverage than a single overly complicated robot [1]. While aerial and terrestrial swarm robotics have been extensively investigated [2,3,4,5], there has been little investigation of underwater robotic swarms. Swarm connectivity is a primary concern of any swarm system, which is realised by intra-swarm communication to enable nodes collaboration. Intra-swarm communication can be achieved in either direct or indirect fashion. Radio and acoustic links are examples of direct communication, whereas indirect communication occurs through the environment, such as stigmergic collaboration [6]. Underwater robotic swarm deployment is particularly challenging, due to the high cost of maritime assets and limited bandwidth of underwater acoustic communication channel. The wide variety of marine missions that can be achieved by means of mobile underwater sensor networks (i.e., underwater swarm robotics), such as deep sea exploration and environmental monitoring, have enabled and motivated underwater robotics research for decades [7]. Localisation is one of the most critical problems in robotic swarms, as it is required to be successfully obtained in advance of nodes’ guidance and control. The navigation module of an autonomous node estimates its position and velocity and then feeds them into the control and guidance modules [8]. In a sub-sea mission, a swarm of mobile and/or static nodes is typically deployed to communicate and collaboratively achieve various predefined tasks in underwater environments. The locations of individual nodes must be known and tracked during operation in order to successfully complete assigned missions. Figure 1 shows a sketch of an underwater wireless sensor network that is represented by a swarm of mobile and static nodes.

Given the absence of the Global Navigation Satellite System (GNSS) in underwater environments, Autonomous Underwater Vehicle (AUV) navigation predominately relies on proprioceptive sensors, such as Inertial Measurement Unit (IMU) integrated with Doppler Velocity Log (DVL) [9]. IMU-based navigation is prone to drift and the DVL is limited to certain ranges i.e., close to seabed [10]. Therefore, acoustic exteroceptive sensors are usually utilised as external navigation aids and integrated with the proprioceptive sensors position estimate in order to reduce the estimated position uncertainty. The severely limited bandwidth and long latency of underwater acoustic communication limit the number of AUVs that can be deployed at once to collaboratively complete a mission [11,12,13]. Underwater multi-agent robotic systems mainly rely on acoustic communication to exchange information among team members with an average propagation speed of 1500 m/s (i.e., speed of sound), with a maximum bit rate of around 60 kbps. On the contrary, information among members of multi-agent terrestrial or aerial robotic systems are exchanged in the speed of light of $3\times 10^{8}$ m/s with bit rate in Mbps.

Advancement in both underwater senors and robotics, together with the rising need for autonomous underwater surveys in deep oceans, have entailed expanding the state-of-the-art in underwater swarm robotics navigation. The navigation framework investigated herein is motivated by the robustness and scalability requirements of an underwater robotic swarms for ocean bottom seismic or similar sub-sea missions. The robustness can be stemmed from four different factors, namely redundancy, decentralisation, simplicity of the individuals, and multiplicity of sensing [1]. Much of the recent research in cooperative multi-agent maritime systems has focused on the provision of path planning algorithms for oceanic field sampling [14,15]. A fleet of three gliders has been deployed for temperature observations in [16] and a fleet of six gliders has been deployed for adaptive sampling and prediction in [15]. In both [15,16], gliders coordinate their motion for ocean sampling, but they rely on radio communication for cooperation when they are on the sea surface. The work presented in [14] considered a team of three AUVs for adaptive ocean sampling, where the problem of reconstructing an oceanic field has been approached as a deterministic optimisation problem. Each node in the team is in favour of covering the area of interest with minimum sampling points while keeping in contact with the rest of the team [14]. Likewise, in [17], the authors proposed a cooperative sampling approach "sampling-on-demand" to support a fleet of underwater gliders to optimise the sampling process with a predefined acceptable uncertainty. A behaviour-based cooperative algorithm for a mobile sensor node was proposed in [18], where each sensor node is mounted on an AUV. The algorithm presented in [18] has been evaluated based on the coverage performance. It is worth mentioning that most of the work in cooperative underwater robotics has considered a heterogeneous maritime assets i.e., a group of sea-surface vehicles collaborating with a few underwater vehicles to complete a mission e.g., oceanic field sampling. Moreover, when a team of AUVs is deployed, they typically keep in contact with a command and control centre in order to facilitate their cooperation [7].

A few articles have addressed the acoustic localisation problem of a cooperative team of a few number of AUVs i.e., 3–4 AUVs. A centralised Extended Kalman Filter (EKF) algorithm was proposed in [19], where the algorithm has access to all sensor data, including range measurements, to reduce the AUV’s location estimate uncertainty. A decentralised approach was proposed in [20], where the Extended Information Filter (EIF) is utilised in order to enhance the performance of the algorithm that was proposed in [19]. The authors in [20] considered a server and client AUVs and showed that the decentralised EIF is able to estimate the client’s state and track the joint probability distribution between the server and the client with a similar performance of the centralised EKF reported in [19] without access to the server’s sensor data. Both [19,20] have considered a single beacon navigation aid (i.e., range measurements) to reduce the AUV’s location estimate uncertainty. Similarly, the authors in [21] employed the EKF for range measurements aid and Time of Flight (ToF) acoustic navigation aid (i.e., Ultra-short Baseline (USBL) fixes) in a network of a USBL, an AUV and two static sensor nodes. A single beacon navigation aid represented in range measurements update between two cooperative AUVs was investigated in [22]. The results presented in [22] showed that the particle filter provides better location estimates of the AUV than the EKF in range-only measurements update. The development of smart mobile sensor network that provides node localisation as a service for an existent acoustic network was discussed in [23]. A fleet of small and low cost AUVs (i.e., ecoSUB [24]) was utilised in [23], where range measurements aided Dead Reckoning (DR) navigation was implemented for node localisation [25]. The authors in [26,27] proposed a confidence-based localisation method for cooperative underwater robotic swarms. The localisation method proposed in both [26,27] strongly relies on localisation methods’ error characteristics that cannot be always accurately obtained in advance. Previously, we have proposed an underwater localisation method to dynamically fuse multiple localisation estimates of an AUV while using fuzzy decision support system in [28]. We sufficed with a simple proof-of-concept simulation and localisation methods’ error characteristics to validate our approach in [28].

In this article, a sophisticated simulation platform is implemented, in which three underwater localisation methods are considered to validate our approach, namely IMU-based DR, trilateration/multilateration [29], and USBL. An industrial grade IMU is modelled and trilateration localisation methods are implemented. A physics-based simulation platform that considers underwater environments hydrodynamics and underwater acoustic communications characteristics was implemented to validate the proposed localisation framework. A physics-based high fidelity robotic simulator Webots [30] was employed to simulate the hydrodynamics properties of underwater environments i.e., density, viscosity, and stream velocity in order to generate external static and dynamic forces. The static and dynamic forces are then applied on the AUVs’ body and the corresponding thrust power is generated for guiding each AUV to its destination. The Underwater Network Project for acoustic simulation (i.e., UnetStack) [31] is employed for underwater acoustic communications simulation, in which the characteristics of the employed channel, modems, Medium Access Control (MAC) protocol, and time synchronisation are all considered. The proposed cooperative navigation framework organises the cooperation among swarm nodes, as exteroceptive sensors navigation aids of each AUV are regulated by the implemented fuzzy rules. The proposed localisation framework utilises fuzzy logic for information fusion, which has inherent advantages over EKF-based fusion, such as design simplicity and flexibility. It is straightforward to capture human expert knowledge in characteristics of localisation methods that are involved in using fuzzy logic. In addition, new knowledge can be acquired and represented in additional fuzzy rules or modifying rules in the proposed localisation framework. In contrast, EKF-based fusion requires dynamic motion models, Gaussian error models, and major changes should be made to accommodate changes in the motion models in the case of integrating additional sensory information. Computational efficiency is another feature that can be gained when adopting fuzzy logic for information fusion over EKF-based fusion. Matrix operations in the EKF involve matrix inverse and multiplication that is computationally expensive for small and dense matrices (i.e., computational complexity of $O(n^{3})$ for n×n matrices), but fuzzy inference, on the other hand, is easy to parallelise in rule evaluation [32]. In addition, fuzzy logic chips for embedded hardware are available for optimised memory demand and the computation speed of fuzzy controllers [33]. The proposed method can be easily extended to accommodate some other newly developed localisation methods by expanding the fuzzy rule base and, thus, better scalability is obtained with increasing swarm size. The proposed method’s localisation performance is compared to USBL-aided DR navigation [34] with round-robin scheduling [35] in extensive simulation with a swarm size of 150.

The remainder of this article is organised, as follows. Section 2 introduces the reader to industrial ToF acoustic navigation systems, inertial navigation system, and compares three methods to solve the trilateration problem. Section 3 illustrates fuzzy logic in the navigation context and the proposed framework. Section 4 explains, in detail, the implemented fuzzy-based localisation algorithm, illustrates the implemented simulation platform, simulation settings, and scenario, and compares the proposed localisation algorithm’s performance to other navigation frameworks i.e., EKF-based navigation with round-robin scheduling. Finally, Section 5 concludes this article.

## 2. Underwater Localisation Methods

The proposed underwater navigation framework considers three localisation methods (i.e., location estimators), namely USBL, IMU-based DR, and trilateration /multilateration.

### 2.1. Time of Flight (ToF) Acoustic Navigation

**Figure 2:**
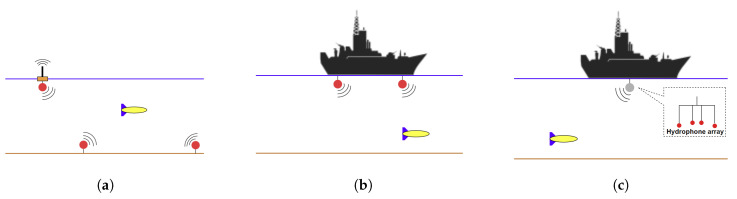
(**a**) Long Baseline (**b**) Short Baseline (**c**) Ultra-short Baseline.

ToF acoustic navigation methods, such as Long Baseline (LBL) and USBL, have dominated the underwater localisation industry since the 1960’s [36]. The LBL is an acoustic localisation system, where the distances among the baseline stations (i.e., transceivers) are long relative to the distance between them and the vehicle i.e., transponder. The baseline stations are fixed at some known positions on the sea surface or on the seabed, as shown in Figure 2a. The vehicle interrogates the baseline station network by broadcasting a signal, and each baseline station would respond back with its location; given the sound speed, range measurements can be obtained by ToF and, therefore, the AUV’s position can be obtained by trilateration.

The Short Baseline (SBL) has the same working principles of the LBL, but the distances among the base stations do not exceed 20–50 m. The base stations are normally mounted on different locations on the operation vessel, as shown in Figure 2b.

The USBL is a hull-mounted system on a surface vehicle or buoy with hydrophones arrays (i.e., transducer) that are separated by very short distances typically 10 cm apart where acoustic signals Time of Arrival (ToA) and phase delays are detected to triangulate a limited number of transponders’ positions within localisation accuracy of 0.13–0.27% of slant range e.g., 10 AUVs can be navigationally aided with an update rate of 1 Hz by a hull-mounted USBL system from Sonardyne [37].

The readers are referred to [38,39] for more details on those systems. The localisation accuracy of ToF acoustic navigation can be further improved through the careful utilisation of filtering or optimisation techniques, such as Kalman filtering [40]. The number of underwater targets that can be localised in ToF acoustic navigation methods is limited and the update rate decreases proportionally with the number of targets due to Time-division multiple access (TDMA) utilisation in network interrogation [36].

### 2.2. Inertial Navigation System

A three-axis gyroscope, three-axis accelerometer, and three-axis magnetometer are often encased in a single chip and referred to as nine-axis IMU. Some of the industrial-grade electronic navigation boards consist of a 9-axis IMU and optionally run Kalman filter to estimate Roll, Pitch, and Yaw angles, as well as to fuse external navigation aids (i.e., from the GNSS) to better estimate the pose of an AUV [41]. The gyroscope returns the AUV’s angular velocity ${}^{i}\omega_{b,t}$ of the body frame b in the inertial frame of reference i corrupted by time-varying bias $B_{t}$ and Gaussian noise $e_{\omega ,t}$ and, therefore, the gyroscope’s row measurements $y_{\omega ,t}$ can be modelled by

(1) $$y_{\omega ,t}={\;}^{i}\omega_{b,t}+B_{t}+e_{\omega ,t}$$

where the gyroscope error $e_{\omega ,t}$ is drawn from Gaussian distribution $N\left(0,\Sigma_{\omega }\right):\Sigma_{\omega }\in R^{3\times 3}$ and the bias $B_{t}$ can be modelled as random walker. Likewise, the accelerometer measures the force ${}^{b}f_{t}$ in the body frame of reference b at each time step *t* and its readings are corrupted by time-varying bias $A_{t}$ (i.e., random walker model) and zero mean Gaussian noise $e_{a,t}$; the raw measurements $y_{a,t}$ of the accelerometer can be modelled by

(2) $$y_{a,t}={\;}^{b}f_{t}+A_{t}+e_{a,t}$$

(3) $${}^{b}f_{t}={\;}^{b}R_{n,t}{(}^{n}a_{t}{-}^{n}g_{t})$$

where ${}^{b}R_{n,t}$ rotates the resultant vector of ${(}^{n}a_{t}{-}^{n}g_{t})$ from the navigation frame n to the body frame b. The accelerometer measurements are dominated by the gravity vector ${}^{n}g_{t}$ and, therefore, the linear acceleration ${}^{n}a_{t}$ can be approximately assumed to be zero

(4) $$y_{a,t}=\;{-}^{b}R_{n,t}{\;}^{n}g_{t}+A_{t}+e_{a,t}$$

On the other hand, the magnetometer complements the accelerometer in order to find the AUV’s heading around the gravity vector i.e., yaw angle. Given the local magnetic field of the earth ${}^{n}m$, the magnetometer raw measurements $y_{m,t}$ can be then modelled by

(5) $$y_{m,t}={\;}^{b}R_{n,t}{\;}^{n}m+e_{m,t}$$

where $e_{m,t}∼N\left(0,\Sigma_{m}\right):\Sigma_{m}\in R^{3\times 3}$ represents the magnetometer measurement noise.

The local magnetic filed of the earth can be accurately determined from geophysical studies [42], as it solely depends on the experiment location. The integration of the gyroscope measurements estimates the sensor’s orientation and the double-integration of the accelerometer measurements estimates the sensor’s position after subtracting Earth’s gravity. The integration process and the noisy measurements of the IMU result in integration drift; the problem of integration drift is even exacerbated when low cost IMU is utilised; therefore, external navigation aids must be fused by e.g. the EKF. The standard implementation of the EKF is adopted for comparisons with the proposed fuzzy-based navigating framework. Interested readers are referred to [43] for more details on IMU modelling, DR navigation, and EKF implementation.

### 2.3. Least-Squares Trilateration

**Figure 3:**
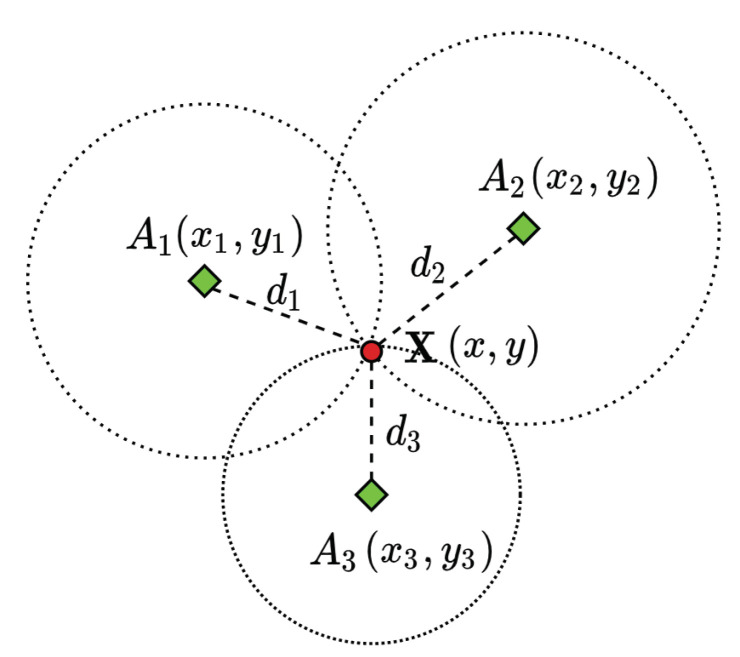
Two-dimensional trilateration problem of determining an object (red solid circle) location X given the location of three stations (green diamonds) $A_{i}$ and the range measurements/distances $d_{i}$ (i=1,2,3).

Locating an object by its range measurements to three reference locations is known as trilateration. The term multilateration is used when there are four or more references. This problem in its simplest form can be interpreted as finding the intersection of four spheres. The three-dimensional localisation problem can be converted into its two-dimensional (2D) counterpart via orthogonal projection [44]. Figure 3 shows a 2D trilateration problem, with no error in range measurements, interpreted as finding the intersection of three circles. A considerable amount of literature has been published on solving the trilateration problem in robotics.

In principle, an object location (x,y,z) can be determined given the location of multiple references $\left(x_{i},y_{i},z_{i}\right)$ and their corresponding range measurements $d_{i}$ by solving a system of equations in the form of Equation (6).

(6) $${\left(x-x_{i}\right)}^{2}+{\left(y-y_{i}\right)}^{2}+{\left(z-z_{i}\right)}^{2}=d_{i}^{2}$$

Closed-form and numerical solutions have been proposed in the literature for solving the trilateration problem. A closed-form solution has been presented by Thomas and Ros in [45], which derives a formula containing a few numbers of Cayley-Menger determinants [46] related to the geometry of tetrahedra. Coope, in [47], presented a rather generic closed-form method to find the intersection points on *I* spheres in $R^{I}$. All closed-form solutions have relatively low computational complexity and they do not accommodate the situation when a solution does not exist. However, numerical methods are more computationally complex, but they estimate the best solution in the case that the unique intersection point of the spheres does not occur due to, for example, range measurements errors. A numerical method, called Taylor series estimation, was presented in [48] to iteratively improve the initial guess by finding the local linear least-sum-squared-error correction. A study by Nadivi et al. compared three statistical methods in the trilateration context, namely linear least-squares estimator, iteratively re-weighted least-squares estimator, and nonlinear least-squares estimator, and showed that nonlinear least-squares estimators perform the best when error in range measurements are considered [49]. In nonlinear least-squares, the problem of trilateration in Equation (6) is re-written as in Equation (7). Equation (7) can be minimised either by local deterministic optimisers, such as Gauss–Newton [50], which requires finding the derivative to minimise the residual of Equation (7) or by global stochastic optimisers, such as Particle Swarm Optimisation (PSO) [51].

(7) $$\min_{X}\sum_{i=1}^{I}{\left(||X-A_{i}{\left|\right|}_{2}-d_{i}\right)}^{2}$$

where ${\left||.|\right|}_{2}$ denotes the Euclidean norm, *I* is the total number of reference nodes, and $A_{i}$ is the position of reference node of index (i) i.e., $A_{i}=\left(\begin{array}{c} x_{i}\\ y_{i}\\ z_{i} \end{array}\right)$ and $d_{i}$ is the corresponding distance between the target position X and $A_{i}$. We seek the coordinates of X i.e., $X=\left(\begin{array}{c} x\\ y\\ z \end{array}\right)$, such that Equation (7) is minimised.

Although local deterministic optimisers, such as Newton’s methods, rely heavily on the initial guess and they do not converge in some cases where singularities may occur i.e. when the references are almost aligned, trilateration has been usually solved by newton’s methods in literature [50]. The Gauss–Newton algorithm [50] starts with an initial guess $X^{\left(0\right)}$ until the cost Equation (7) is iteratively minimised. Given the residual functions $r_{i}\left(X\right)=||X-A_{i}{\left|\right|}_{2}-d_{i}$, Gauss–Newton proceeds by the iterations, as follows

(8) $$X^{\left(s+1\right)}=X^{\left(s\right)}-{\left(J_{r}^{⊤}J_{r}\right)}^{-1}J_{r}^{⊤}r\left(X^{\left(s\right)}\right)$$

where the superscript (s) is the iteration index, $r\left(X\right)=\left(\begin{array}{c} r_{1}\left(X\right)\\ \vdots \\ r_{I}\left(X\right) \end{array}\right)$ is the vector of residuals, the symbol ${}^{⊤}$ denotes matrix transpose, and $J_{r}$ is the Jacobian matrix of r(X). This iterative process is carried out until either the maximum number of iteration is reached or no further improvement is achieved i.e., $\left|\right|X^{\left(s\right)}-X^{\left(s-1\right)}{\left|\right|}_{2}$ is lower than a predefined threshold.

In general, global stochastic optimisers tend to perform better in nonlinear least-squares problems, but they are computationally expensive. PSO starts with randomly initialising a set of candidate solutions (particles) over the search space, and each particle keeps updating its best experience (Pbest) over the objective function and the algorithm keeps updating a record of the entire swarm’s best experience (Gbest) over the objective function. The velocity and the position of each particle *j* are then determined in every iteration based on (Pbest) and (Gbest) according to the following formula

(9) $$V_{j}^{\left(s+1\right)}=\omega V_{j}^{\left(s\right)}+c_{1}r_{1}\left(P{\mathrm{best}}_{j}^{\left(s\right)}-X_{j}^{\left(s\right)}\right)+c_{2}r_{2}\left(G{\mathrm{best}}^{\left(s\right)}-X_{j}^{\left(s\right)}\right)$$

(10) $$X_{j}^{\left(s+1\right)}=X_{j}^{\left(s\right)}+V_{j}^{\left(s+1\right)}$$

where (s) is the iteration index, $c_{1}$ and $c_{2}$ are two positive constants, $r_{1}$ and $r_{2}$ are two randomly generated numbers ∼unif(0,1), ω is the inertia constant, such that ω=0.9−(0.005s), Pbest is the best position particle based on its own experience, and Gbest is the global best position particle based on the entire swarm’s experience. The constants $c_{1}$ and $c_{2}$ are typically set to two, as per the common practice of PSO [52]. In PSO, each particle represents a candidate solution and particles are randomly generated over the search space at the first iteration. Each particle $X_{j}^{\left(s\right)}$ is evaluated at the iteration (*s*) on the objective function i.e., Equation (7). Pbest and Gbest are updated at each iteration accordingly. Each particle’s velocity and position are then updated, as in Equations (9) and (10), respectively.

Zhou, in [53], proposed an algorithm with low computational complexity and high operational robustness for solving the nonlinear least squares trilateration/multilateration problem in a closed-form solution while using standard linear algebra techniques. In other words, the algorithm in [53] can find the best approximation in a closed-form solution, even if the solution cannot be found using others closed-form methods due to errors in range measurements. The nonlinear least squares trilateration/multilateration problem in Equation (7) can be rewritten as

(11) $$\min_{X}\sum_{i=1}^{I}{\left({\left(A_{i}-X\right)}^{⊤}\left(A_{i}-X\right)-d_{i}^{2}\right)}^{2}$$

The author in [53] mathematically proved that the optimal X in Equation (11) can be obtained from the following linear Equation

(12) X=q+c

where c and q vectors i.e., $q=\left(\begin{array}{c} q_{1}\\ q_{2}\\ q_{3} \end{array}\right)$ can be found by Equations (13) and (14)

(13) $$c=\frac{1}{I}\sum_{i=1}^{I}A_{i}$$

(14) $$q_{1}^{2}+q_{2}^{2}+q_{3}^{2}=-\frac{1}{I}\sum_{i=1}^{I}A_{i}^{⊤}A_{i}+\frac{1}{I}\sum_{i=1}^{I}d_{i}^{2}+c^{⊤}c$$

Interested readers are referred to [53] for more details on obtaining the vector q and for the mathematical derivation.

The three methods are implemented and compared in order to solve the least-squares multilateration problem i.e., Gauss–Newton [50], PSO [51] and analytical closed-form approach [53]. A Monte-Carlo simulation of 100 random walkers in a confined region of 100 m${}^{3}$ is considered in order to compare the three aforementioned nonlinear least-squares solvers for the multilateration problem. If any three or more walkers are in the communication range of 25 m of another walker, trilateration/multilateration is performed with zero error in range measurements. The following histograms compare the accuracy of each method, i.e. Gauss—Newton (local gradient-based optimiser), PSO (global stochastic optimiser) and analytical approach presented in [53] in solving the least-squares trilateration/multilateration problem.

**Figure 4:**
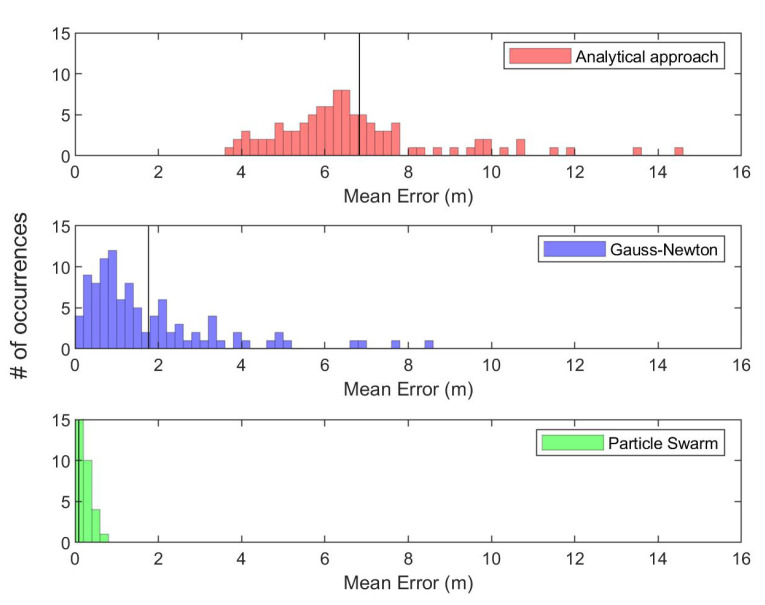
Histograms of mean multilateration error of around 15,000 multilateration process carried out in 100 walkers (nodes); the vertical line in each histogram represents the mean error of the entire simulation.

The three aforementioned methods have been adopted to solve the least-squares trilateration/multilateration problem on the exact same network topology for each simulation step. The trilateration/multilateration process has been carried out around 15,000 times for each method/optimiser. Figure 4 clearly shows the superiority performance of PSO [51] over the analytical approach [53] and Gauss–Newton [50] in solving the multilateration problem. The entire swarm mean localisation error when the analytical approach [53], Gauss–Newton [50] and PSO [51] are adopted is 6.82 m, 1.76 m and 0.089 m, respectively. There is an improvement of 74% in the mean trilateration error when the Gauss–Newton optimiser is compared to the analytical approach presented in [53] and an improvement of 94% when PSO is compared to Gauss–Newton. Therefore, PSO is adopted hereafter to solve the least-squares trilateration/multilateration problem.

## 3. Fuzzy-Based Localisation

Fuzzy Logic [54] is efficient in information fusion, especially for uncertain or conflicting information. On the contrary, the family of Gaussian filters [55], such as EKF or Unscented Kalman Filter (UKF), requires Gaussian modelling of information uncertainty. Information fusion problem in fuzzy logic is transformed into simple input-output mapping. Many other systemsm such as neural networks, differential equations, and lookup tables, can be used for input-output mapping, but fuzzy logic is still the most convenient method. Fuzzy logic is adopted in robotic swarms literature in order to mitigate various challenges due to its simplicity. The authors in [56] adopted a fuzzy logic-based voter to decide on an AUV’s health (i.e., the AUV is either fit for the task or must go back to the base) in swarm behaviour simulation of multiple AUVs. Fuzzy logic can be seen as a method for computing with words instead of numbers and, thus, it is convenient to use as it imitates human reasoning skills. It is the codification of common sense and, therefore, a lot closer to human intuition than any other input-output mapping method.

**Figure 5:**
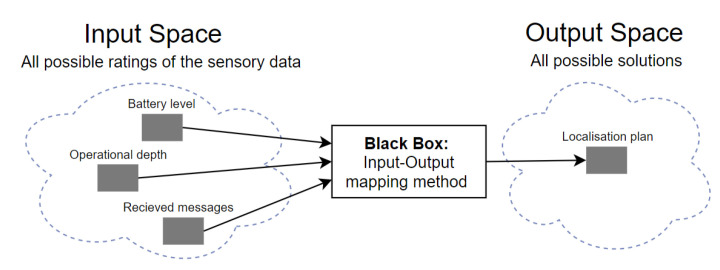
An example of input-output mapping for the localisation problem: “Given three variable inputs what the localisation plan should be?”

An example of input-output mapping for the underwater localisation problem that is based on three linguistic variable inputs, i.e., the operational depth, battery level, and received messages, is shown in Figure 5. Each variable input is associated with several ordinal or categorical linguistic concepts with vague boundaries. For example, the operational depth can be described by different adjectives, like shallow, deep and very-deep. These adjectives are the labels of linguistic concepts modelled by fuzzy sets [54]. In contrast, the classical classification in crisp sets would either include an input in one set or exclude it. A membership function has to be associated with each fuzzy set to map an input of the entire input space, universe of discourse, to its membership value between 0 and 1 [54].

If-then rules are the main constituent of fuzzy logic systems, they combine the antecedent (if-part) with the consequent (then-part) [32]. All variable inputs in the antecedent are resolved to a graded membership between 0 and 1 (uzzification), but if the antecedent has multiple parts, fuzzy logic operators such as AND (minimum) and OR (maximum) are applied to resolve the antecedent to a single number between 0 and 1, which is the degree of support for the rule (application) [57]. The resultant degree of support of the rule is then used to shape the output fuzzy set (implication) [57]. All if-then rules are being processed in parallel, so that the order of the rules does not make a difference. Each if-then rule results in a fuzzy set, all resultant output fuzzy sets are combined to give a single resultant fuzzy set by taking the maximum or any other methods, such as sum of the rule output sets (aggregation) [54]. Given the aggregation process output fuzzy set and the output space of the output fuzzy set, the resultant aggregate output fuzzy set is converted into a single number (defuzzification) [54].

One example of an IF-THEN fuzzy linguistic rule considering the localisation problem:

IF Operational Depth is Shallow AND Battery Level is High THEN Localisation Method is L.

where Operational Depth and Battery Level are two variable inputs, Shallow and High are two fuzzy sets for describing the inputs, both parts of the antecedent are combined by AND logical operator, and Localisation Method is the variable output where L is its fuzzy set that refers to a particular localisation method and to be reshaped based on the rule degree of support.

Assume a swarm of AUVs is launched from known positions on the sea surface and a USBL system, which can navigationally aid only a limited number of AUVs in each of its TDMA frame. Each AUV is equipped with long/medium Acoustic Communication (ACOMMS) modem (USBL transponder), short range ACOMMS modem for intra-swarm communication, nine-axis IMU, and depth sensor. Assume that we have *n* underwater localisation methods. Each method can localise an underwater mobile sensor node with best accuracy under certain operational conditions e.g., AUV’s operational depth and the reception of acoustic messages. This approach allows for each AUV to either select a single localisation method or to fuse two or more localisation methods’ estimates to improve localisation accuracy by increasing localisation aid updates along the whole trajectory based on fuzzy inference system. Mamdani fuzzy logic is adopted for the localisation problem due to its intuition and well-suitability to human input, which can be easily captured while using if-then rule construct [58]. Moreover, the impreciseness of human expert knowledge can be modelled and processed by fuzzy inference.

**Figure 6:**
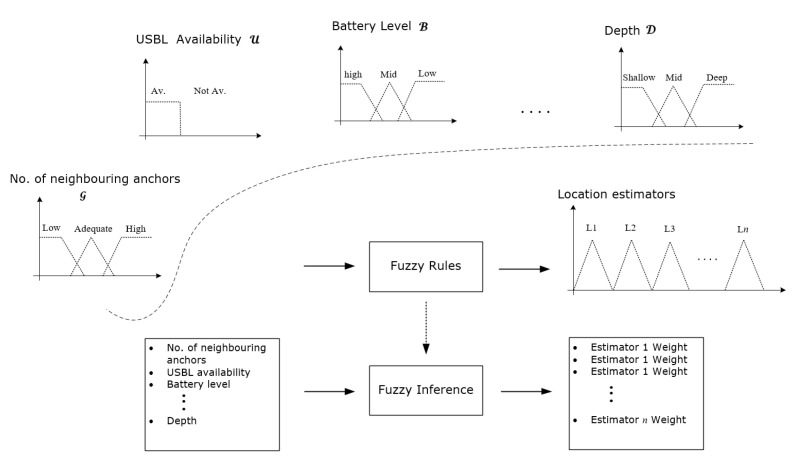
Fuzzy-based localisation.

Figure 6 illustrates four examples of decision support elements (input variables) for this approach, namely operation depth D, USBL availability U, AUV’s battery level B, and number of localisation aids received within a predefined time window from neighbouring AUVs G. Input fuzzy sets are determined intuitively based on the features of the variable inputs e.g., a USBL fix is either received by the AUV’s transponder or not received. The final location estimate could be the output of either a single location estimator or a weighted combination between two or more location estimators based on the normalised sum of the firing strength in each of the output fuzzy set. Each considered localisation method is represented by a fuzzy set of a disjoint triangle over the universe of discourse, so that each localisation method contribution in the final localisation plan can be easily obtained by its firing strength.

## 4. Simulation

### 4.1. Simulation Platform

Underwater robotic swarm simulation e.g., [56] is able to provide a cost-effective way to evaluate the system performance (e.g., localisation accuracy and scalability) of large underwater robotic swarms. Intra-swarm communication is the backbone of cooperative navigation algorithms; therefore, a realistic underwater acoustic simulation is considered. Robots’ physics and environment’s hydrodynamics can be simulated on Webots, but underwater acoustic communications properties cannot be simulated on Webots or any other physics based robotic simulators, such as Gazebo [59] and CoppeliaSim [60]. Therefore, the Underwater Network Project for acoustic simulation (i.e., UnetStack) [31] is employed for underwater acoustic communications simulation. UnetStack is an agent-based network stack and simulator developed to support highly optimised protocols for underwater acoustic sensor networks [31]. It allows for easy network configurations and management and it allows protocols to be simulated in realistic channel conditions. The nature of underwater acoustic communication requires significant cross-layer information sharing. Howeverm most, if not all, network simulators are not design for cross-layer sharing. For example, the network simulator ns2 [61] was not originally implemented for cross-layer sharing. However, an extension was released (i.e., *miracle*) [62] to allow cross-layer information sharing. Underwater acoustics simulators, such as DESERT [63] and SUNSET [64], are ns2-based and of course they made use of miracle plugin [62]. UnetStack, however, adopted service-oriented agent architecture that fundamentally allows for cross-layer interaction. UnetStack supports both discrete-event simulation mode and real-time simulation mode. Therefore, the developed protocol can be deployed to any compatible underwater communication modem, such as Evologics [65], for field experiments directly without the need for recompilation.

The acoustic channel model with Urick [66] acoustic and Binary Phase-Shift Keying (BPSK) fading [67] models are utilised in our UnetStack simulation. The UnetStack Urick acoustic model is parameterised by water depth, temperature, salinity, bandwidth, carrier frequency, spreading loss factor, and noise power spectral density level. Sound speed in this model is computed based upon the nine terms equation that was proposed by Mackenzie in [68] and transmission loss is computed as in [66]. Given the transmission source level $S_{L}$, the computed transmission loss $T_{L}$ and the predefined noise level $N_{L}$, the Signal-to-Noise Ratio (SNR) in **dB** re μPa **@**1 m would be $S_{L}-T_{L}-N_{L}$.

The BPSK fading model uses Urick acoustic model’s SNR to simulate a frame’s detection and successful decoding. Rician fading parameter, fast or slow fading, acceptable probability of false alarm during detection, and processing gain parametize the UnetStack BPSK fading model. If fast fading is enabled, the error in each bit is generated in the simulation independently from Rician fading model with Gaussian noise. In contrast, a single realisation of Rician fading model with Gaussian noise is added to the entire frame when slow fading is enabled instead. The UnetStack simulator is explained in details and validated through field experiments in [69].

**Figure 7:**
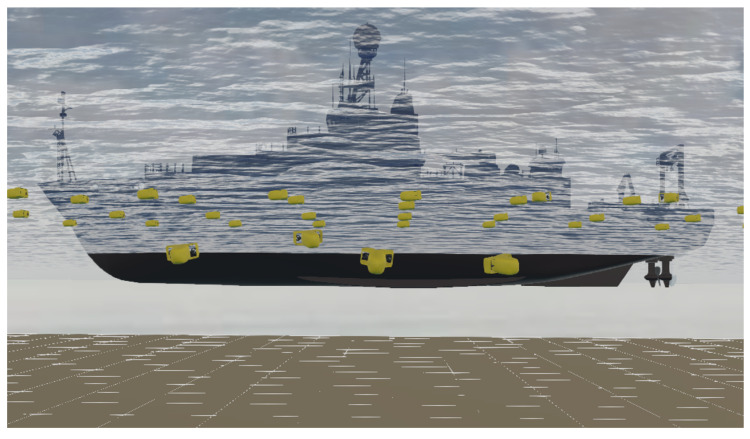
Underwater Webots simulation scene of 50 AUVs deployment, the USBL transceiver is hull-mounted on the deployment vessel.

A swarm of identical AUVs and static deployment vessel are simulated on Webots; each AUV is subject to static (i.e., Archimedes’ thrust) and dynamic (i.e., drag) forces exerted by the simulated fluid properties on Webots, such as density, viscosity, and stream velocity [30]. Each AUV in the swarm has its own controller, which is written by Webots Matlab Application Programming Interface (API)s. Figure 7 shows a Webots scene example of AUVs swarm deployment in shallow water and the deployment vessel.

A Webots supervisor-enabled node (Sniffer) is allocated to monitor each AUV in the swarm. If an AUV broadcasts a navigation aid, the Sniffer node reports the network topology to UnetStack, runs the simulation, and reports back the results to each AUV in the swarm in terms of received messages content (if any) and time of arrival. The centralisation of the USBL system and its TDMA frame and slots are simulated by a Webots supervisor-enabled node, as the proposed algorithm requires the USBL to respond to the AUVs in first-come-first-served basis when localisation requests received by the USBL are more than can be aided in a single TDMA frame.

**Figure 8:**
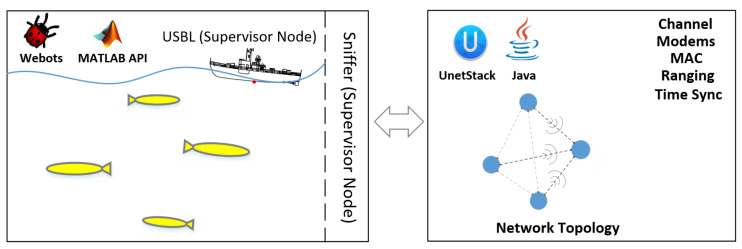
The implemented simulation platform used to validate the proposed navigation algorithm. Webots robotic simulator is employed for physics simulation and UnetStack is employed for simulating the underwater acoustic communication properties.

Figure 8 shows an example simulation scenario of four AUVs navigating to their destinations and only three of them broadcast localisation messages, the “Sniffer” reports the network topology to UnetStack simulator and reports back the results of localisation aids delivery and time of arrival to the corresponding AUVs.

**Figure 9:**
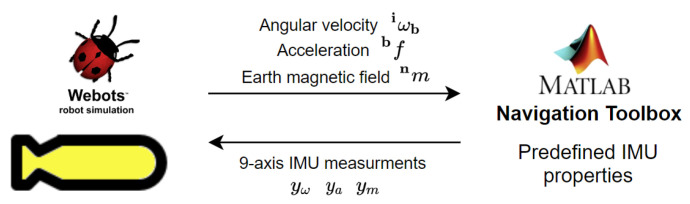
Angular velocity, acceleration, and local magnetic field of the earth are generated as ground truth readings at each time instant of each IMU that is modelled in an underwater environment in Webots simulator. Given the ground truth readings and the IMU properties, a realistic nine-axis IMU is modelled on MATLAB Navigation toolbox.

A typical industrial grade IMU is considered in our simulation [41] for DR navigation. Webots robotic simulator provides a nine-axis IMU sensor model in order to return the AUV’s roll, pitch, and yaw angles with respect to the world coordinate, the AUV’s acceleration, and angular velocity can be also obtained [30]. Webots utilises a lookup table to add error models to the IMU measurements or to match Webots IMU’s output with device specific output; this look-up table can be very hard to generate, given the IMU’s model complexity. However, MATLAB Navigation toolbox seamlessly models all of the intricacies of a nine-axis IMU with predefined properties, such as velocity and angle random walks, bias instability, axis misalignment, and constant bias. Therefore, we generate the angular velocity ${}^{i}\omega_{b,t}$, the acceleration ${}^{b}f_{t}$, and the local earth magnetic field ${}^{n}m$ of Webots inertial unit sensor with no noise or biases added and feed them into MATLAB Navigation toolbox for nine-axis IMU sensor modelling. Figure 9 shows a diagram of the procedure that we followed to model a nine-axis IMU.

### 4.2. Implementation

Assume that each AUV is equipped with long/medium ACOMMS modem (USBL transponder) working at low frequency band i.e., 20–40 kHz, short range ACOMMS modem working at high frequency band (i.e., 100–180 kHz) for intra-swarm communication, nine-axis IMU, depth sensor, and Chip Scale Atomic Clock (CSAC) for clock-synchronisation of the AUVs [70]. Range measurements are acquired by means of One-Way Travel Time (OWTT) [71]. Timestamps for range measurements are subject to Gaussian additive noise of zero mean and standard deviation of 1.2 ms [72], which corresponds to error standard deviation of 1.8 m in range measurements given that the average speed of underwater acoustic waves is 1500 m/s. The three-dimensional localisation problem can be converted into its 2D counterpart via orthogonal projection [44], as all AUVs are equipped with pressure sensors for accurate depth estimation. Because of the severely limited bandwidth and high latency in ACOMMS, only a subset of the swarm (i.e., Navigation Beacon (NB)) is made capable of broadcasting navigation aids within a short period of time once any reliable location estimator (e.g., USBL or trilateration) is dominating the final localisation plan. Five variable inputs have been considered in order to determine the weights of the different underwater location estimators in the final localisation plan and they are: USBL availability U, the number of localisation aids received within a predefined time window from NBs G, battery level B, operational depth D, and DR time R. The USBL availability determines whether a USBL fix from the USBL transceivers (on the sea-surface) has been received or not. DR time represents the time period that the AUV has been relying on IMU-based DR for navigation and it resets to zero once a USBL fix or a trilateration/multilateration estimate has been fused. Inputs fuzzy sets have been determined intuitively based on its features e.g., a USBL fix is either received by the AUV’s transponder or not received and, thus, the USBL availability U is represented by crisp sets over the universe of discourse i.e., available or unavailable. In this implementation, the number of localisation aids that is received from NBs is either enough or not enough to perform trilateration i.e., three or more localisation aids received from recent USBL-localised NB AUVs.

**Figure 10:**
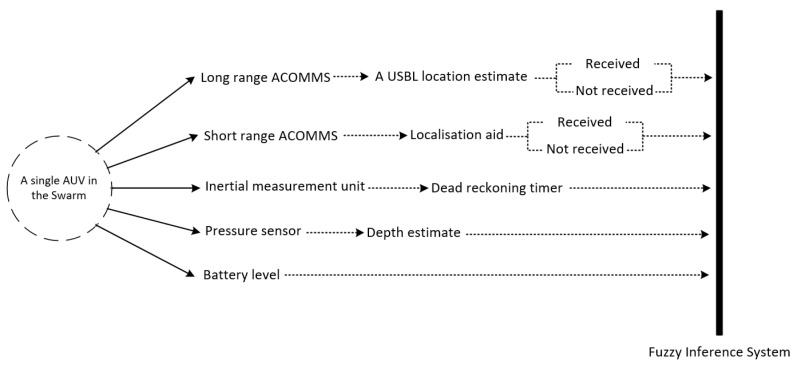
Fuzzy inference variable inputs of each AUV in a swarm of AUVs based on its on-board acoustic communication modems and sensors.

**Figure 11:**
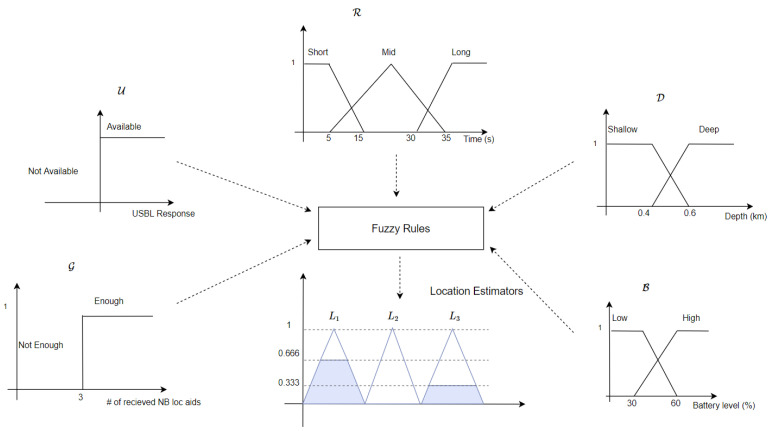
Fuzzy-based underwater localisation approach (Decision-making) with five variable inputs & their fuzzy/crisp sets and three underwater location estimators. The aggregated fuzzy output shows an example of a final localisation plan of $\left(\frac{2}{3}L_{1}+\frac{1}{3}L_{3}\right)$.

Three localisation methods (i.e., location estimators) have been considered in our implementation, namely IMU-based DR, USBL, and trilateration/multilateration, and denoted by $L_{1}$, $L_{2}$, and $L_{3}$, respectively. The final location estimate could be the output of either a single location estimator or a weighted combination between two or more location estimators based on the normalised sum of the firing strength in each of the output fuzzy set. Each of the localisation methods is represented by a fuzzy set of a disjoint triangle over the universe of discourse, so that the firing strength of each localisation method can be easily dissociated and normalised in the final localisation plan. Figure 10 shows fuzzy inference system’s variable inputs that are based on each AUV’s on-board sensors and Figure 11 shows the variable inputs and their fuzzy/crisp sets, their types, and limits, and the three underwater location estimators used in this implementation. An example of a final localisation plan of a combination between $L_{1}$ and $L_{3}$ is shown in Figure 11, where the final location estimate would be $\left(\frac{2}{3}L_{1}+\frac{1}{3}L_{3}\right)$ as $L_{1}$ firing strength is as double as $L_{3}$. Fuzzy set parameters were fine tuned in our simulation based on trial-and-error simulation, but their initial values were estimated based on prior knowledge of each localisation method operating conditions e.g. USBL localisation error is low at shallow operational depths and battery consumption in trilateration localisation is relatively high. Therefore, USBL localisation is most likely to be adopted (if available) at shallow operational depths and trilateration is most likely to be performed if the battery level is high. Appendix A.1 shows the employed fuzzy rule base.

**Figure 12:**
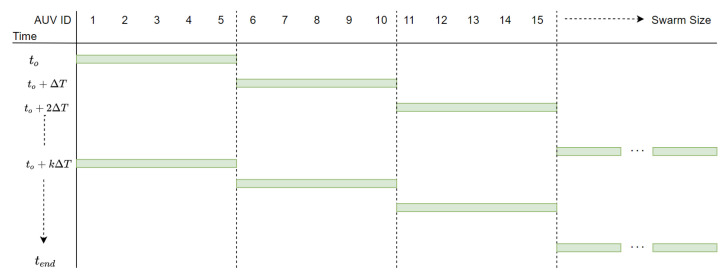
An Example of round-robin scheduling for USBL navigation aid (represented by the green bar) in a swarm of AUVs. The USBL, in this example, can only navigationally aid five AUVs in a single TDMA frame of ΔT.

The proposed underwater swarm localisation algorithm’s performance is compared to USBL-aided DR navigation [34] with round-robin scheduling [35]. It is worth mentioning that, in the proposed fuzzy-based localisation method, if USBL transceivers receive localisation requests from more than the maximum that it can be aided in a single TDMA frame, the USBL employs round-robin scheduling [35] to respond to all AUV’s localisation requests. A subset of the swarm (i.e., NBs) is configured to broadcast navigation aids within a predetermined period of time once any reliable location estimator (e.g., USBL or trilateration) is dominating the final localisation plan. The NB AUVs are a fixed set of AUVs and they are randomly selected in the swarm before deployment. Figure 12 shows an example of round-robin scheduling of a swarm of AUVs for USBL localisation where only 5 AUVs can be localised in a single TDMA frame ΔT. Each subset of five AUVs in Figure 12 can be navigationally re-aided by the USBL after the last node is aided e.g. the first subset of five AUVs is navigationally re-aided by the USBL every kΔT, where *k* is the swarm size divided by the maximum number of AUVs that can be aided in a single TDMA frame e.g., five AUVs. AUVs grouping for round-robin scheduling is performed based on first come, first served basis. In the proposed algorithm, when a NB receives a USBL fix, it broadcasts localisation aids of its North and East coordinates to its neighbouring AUVs. Similar to the MAC protocol that was adopted in GSM communications (i.e., a combination between both Frequency-division multiple access (FDMA) and TDMA [73]), we assume that either FDMA or Code Division Multiple Access (CDMA) MAC protocol [74] is utilised to separate intra-swarm communication from USBL communication and within both intra-swarm and USBL communications TDMA MAC protocol is adopted.

**Figure 13:**
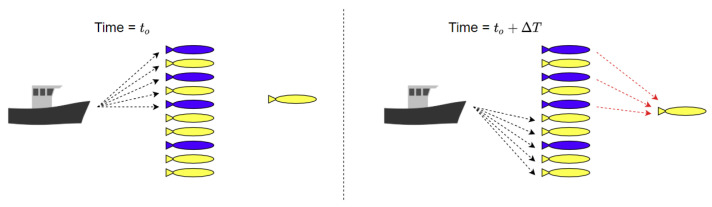
The proposed fuzzy-based localisation framework harnesses round-robin scheduling for USBL navigation aid assuming that all AUVs requested USBL navigation aid. The USBL can only navigatioanlly aid five AUVs in a single TDMA frame of ΔT utilising low-frequency ACOMMS (in black arrows). NBs (in blue) broadcast localisation aid to their neighbouring AUV utilising high-frequency ACOMMS (in red arrows).

Figure 13 shows an example scenario of the proposed algorithm assuming that the USBL received localisation requests from all 11 AUVs that are shown in Figure 13. Given that the USBL can send navigation aids to only five AUVs in a single TDMA frame of ΔT, round-robin is then adopted for USBL navigation aids in three TDMA frames where at $time=t_{o}$ the first subset of five AUVs is aided by the USBL and at $time=t_{o}+\Delta T$ the second subset of five AUVs is aided. Subsequently the third subset of 1 AUV is aided. However in the proposed fuzzy-based localisation method, while the second subset of AUVs is aided by the USBL at $time=t_{o}+\Delta T$, NBs that have been aided by the USBL at $time=t_{o}$ broadcast localisation aid to their neighbouring AUV for trilateration.

### 4.3. Simulation Scenario and Settings

A swarm of N AUVs is launched from known positions on the sea surface, each AUV has a unique ID that is associated with a specific seabed destination. We assume that an AUV’s battery level follows a typical discharging profile of a lithium battery cell [75]. A hull-mounted USBL system on the sea surface can localise 10 AUVs in each of its TDMA frame [37].

A finite state machine with a proportional–derivative controller was designed to guide each AUV to its destination on the seabed through the shortest path i.e., straight line. The AUV’s target yaw and pitch are updated once an external navigation aid is fused. A swarm size of 50 to 150 AUVs has been simulated on Webots. Ten AUVs can be navigationally aided by the USBL in its TDMA frame of 1 s [37], so that a different subset of the swarm can be aided by the USBL every USBL TDMA frame. A fixed subset of the swarm (i.e., NBs) is configured to broadcast localisation aids to their neighbouring AUVs within a predefined period of time, once they are externally aided by the USBL or NBs i.e., the weight of the USBL or trilateration estimators is greater than 0.8 in the final localisation plan of the proposed fuzzy-based localisation method. We consider a short period of time (i.e., 1 s), depends on DR accuracy, in which a NB can broadcast localisation aids once their final localisation plan is dominated by the USBL or trilateration. The same period of time (i.e., 1 s) is considered in the round-robin EKF-based localisation for the NBs to broadcast localisation aids once a USBL fix is received. Table 1 summarises the specifications of the modelled IMU. A subset of 10 AUVs can be navigationally aided by the USBL every 1 s (i.e., USBL TDMA frame length), but we instead delay the USBL update for three more seconds to account for larger swarm sizes (i.e., three times as large) in which the simulation becomes intractable using the available computing resources (Dedicated workstation with Intel Xeon Gold 6148 CPU @ 2.40 GHz 20 Cores, 192 GB RAM, and Nvidia TITAN Xp 12 GB). Table 2 summarises simulation settings and parameters we have considered in our simulation and Table 3 lists the parameters of the simulated intra-swarm ACOMMS.

### 4.4. Results and Analysis

**Figure 14:**
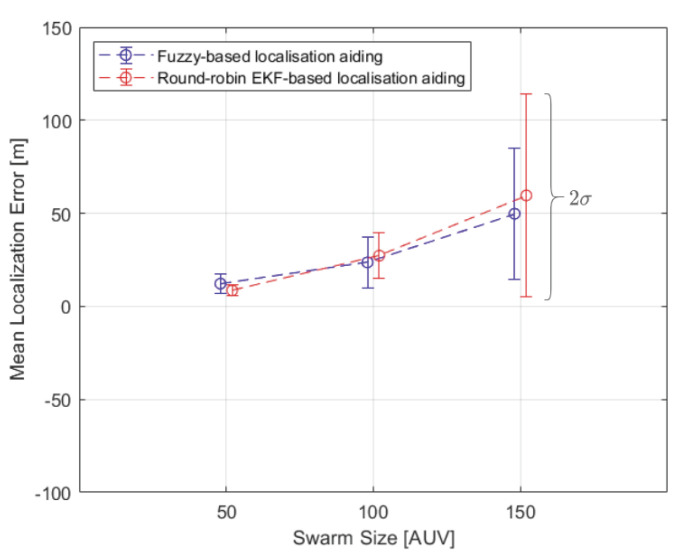
The entire swarm mean and standard deviation localisation error in both the proposed Fuzzy-based USBL/trilateration aided DR navigation (in blue) and round-robin EKF-based USBL/trilateration aided DR navigation (in red). The error bar around the mean point represents 2σ standard deviation.

Swarm sizes of 50 to 150 AUVs are considered to validate and compare the proposed fuzzy-based localisation algorithm’s performance. The performance of each localisation method is compared based on mean localisation error and standard deviation of each AUV, as well as of the entire swarm. Figure 14 shows the entire swarm mean localisation error and standard deviation in each simulation trial. Ten NBs are considered in both the proposed fuzzy-based localisation and EKF-based localisation. Round-robin EKF-based aiding at swarm size of 50 outperforms the proposed algorithm in the entire swarm mean localisation error by 29.8%. However the proposed fuzzy-based localisation algorithm outperforms Round-robin EKF-based aiding at swarm size of 100 and 150 AUVs by 13.25% and 16.53%, respectively. The proposed fuzzy-based localisation algorithm greatly improves the entire swarm standard deviation by 35.17% at swarm size of 150 AUVs when compared to the round-robin EKF-based method. One-tail two sample *t*-test is conducted to compare the localisation accuracy performance of each simulation trial in both round-robin EKF-based method and the proposed fuzzy-based method. The null hypothesis is rejected at 0.05 significance level with *p*-values of 0.025 and 0.032 at swarm sizes of 100 and 150 AUVs, respectively. Based on the one tail test, the mean localisation error of the proposed fuzzy-based method is lower than that of the EKF-based method at a significance level of 0.05. Therefore, the mean localisation error of the proposed fuzzy-based method is lower than that of the EKF-based method when swarm size increases. Table A1 in Appendix A.2 shows *p*-values, degree of freedom, t-statistics, and critical values of each simulation trial to statistically compare the localisation accuracy of the proposed fuzzy-based method with the EKF-based method. Figure 14 shows that the break-even point for the proposed algorithm to outperform round-robin EKF-based aiding is at swarm size of around 80 AUVs.

**Figure 15:**
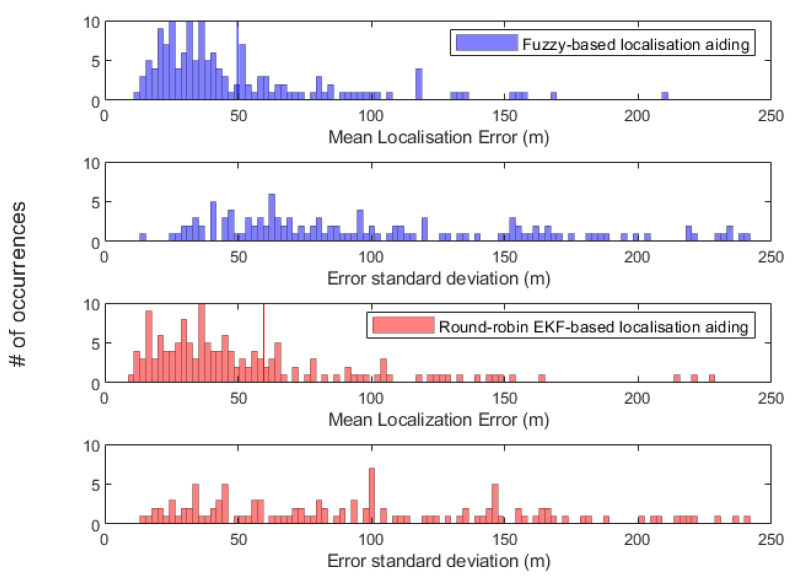
Histogram of the mean localisation error and standard deviation of each AUV of the entire swarm of 150 AUVs. The vertical lines represent the entire swarm mean localisation error, which is 49.76 m and 59.62 m in fuzzy-based and round-robin EKF-based localisation, respectively.

**Figure 16:**
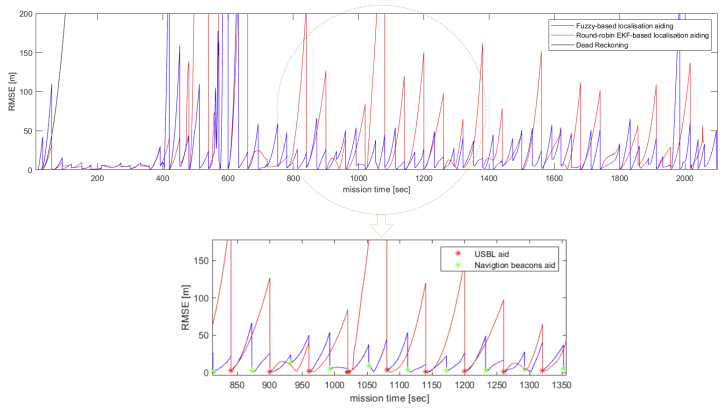
Instantaneous localisation performance of an AUV in a swarm of 150 when the proposed fuzzy-based, round-robin EKF-based and DR-only methods are adopted. A time window of 500 s shows the fused external navigation aid.

Figure 15 shows histograms of mean localisation error and standard deviation of each AUV in a swarm of 150 AUVs. The results presented in Figure 15 are depicted by computing the mean and standard deviation of each AUV along the followed trajectory from its home position on the sea surface to its destination on the seabed. It can be seen that the number of occurrences of standard deviation below 100 m is higher in the proposed algorithm than in round-robin EKF-based localisation. There are 81 out of 150 AUVs achieved standard deviation below 100 m along their trajectories, as opposed to 73 AUVs when round-robin EKF-based aiding is adopted. Localisation performance of an AUV in a swarm of 150 when the proposed fuzzy-based, round-robin-based and DR-only are shown in Figure 16. The AUV’s localisation performance in Figure 16 is aided by the USBL around the same time in both round-robin EKF-based and fuzzy-based aiding and that happened for two reasons. Firstly, the AUV has been exposed to the same environment settings when either of the localisation methods is adopted. Secondly, the fuzzy rules are designed to prioritise a USBL fix whenever it is received.

Figure 16 shows that the AUV’s localisation error quickly accumulates over 200 m in the first 100 s of the mission when there is no external navigation aid available, as in DR-only (black curve). It can be observed that the AUV is externally aided by the navigation beacons more frequently when the proposed fuzzy-based algorithm is adopted.

## 5. Conclusions

In conclusion, design simplicity and flexibility are emphasised in the proposed localisation framework, as new knowledge can be acquired and represented in additional fuzzy rules or modifying existent rules in the proposed localisation framework. In contrast, substantial efforts are needed when integrating new localisation methods in an existent EKF-based navigation for two reasons: Gaussian error modelling; and, the entire filter re-implementation to expand the covariance matrix and the state vector. The proposed method can be easily extended to accommodate different localisation methods, such as DVL aided navigation, by expanding the fuzzy rule base and, thus, better scalability is obtained with increasing swarm size. The proposed fuzzy-based localisation method is computationally less expensive than the EKF-based localisation method, as matrix operations in the EKF that involve matrix inverse and multiplication are computationally expensive. Matrix operations in the EKF is not efficient to parallelise for small matrices (e.g., 6×6 matrices). However, fuzzy inference can be easily parallelised and fuzzy logic chips are available for optimised memory demand and computation speed implementation. The proposed algorithm enhanced the overall localisation accuracy of the entire swarm by more frequently providing the AUVs with external navigation aids. The proposed fuzzy-based aiding has improved the entire swarm mean localisation error and standard deviation by 16.53% and 35.17%, respectively at swarm size of 150 AUVs when its compared to round-robin EKF-based USBL/trilateration-aided DR navigation. The total number of AUVs that achieved a standard deviation below 100 m along their trajectories has increased by around 10% when the proposed fuzzy-based aiding is adopted as compared to round-robin EKF-based aiding. Simple fuzzy rules that capture human expert knowledge in underwater localisation methods by if-then rules are proposed and the impreciseness of expert knowledge is modelled and processed while using fuzzy inference. The proposed algorithm performance is emphasised in large swarm sizes, as it becomes nearly impossible to navigationally aid all AUVs by round-robin scheduling.

In the future, uncertainty indicator of the localisation process will be developed to prioritise the USBL navigation aids for some AUVs over the rest. This uncertainty indicator can also be used to control NBs broadcasting period e.g., when greater than a predefined threshold. The self-tuning fuzzy inference approach will also be designed for adjusting fuzzy set parameters and improving fuzzy rule base by evaluating the algorithm’s response at real time. The proposed localisation framework considers OWTT for range measurements among the AUVs and utilizes the TDMA for cooperative localisation, as all AUVs are assumed to be accurately synchronised. However, it is always possible that a few AUVs’ clocks fall out of synchronisation. Therefore, adopting Two-Way Travel Time (TWTT) for ranging and self-organising TDMA that does not rely heavily on time synchronisation can possibly improve the localisation performance in such cases. 

## Figures and Tables

**Table 1:** Ellipse two micro IMU specifications.

Parameter	Value
Accelerometer Resolution	60.958 μg
Accelerometer Constant Bias	14 μg
Accelerometer Noise Density	57 μg/$\sqrt{Hz}$
Gyroscope Resolution	0.0625${}^{\circ }$
Gyroscope Constant Bias	7${}^{\circ }$/hour
Gyroscope Noise Density	0.15${}^{\circ }$/$\sqrt{\mathrm{hour}}$
Magnetometer Resolution	1 mGauss
Magnetometer Constant Bias	1.5 mGauss
Magnetometer Noise Density	3 mGauss

**Table 2:** Simulation parameters.

Parameter	Value
Swarm Size	50; 100; 150 AUVs
Simulation Time Step	100 ms
Clock-synchronisation error	1.2 ms 1-σ
Seabed Depth	1000 m
Depth Sensor	2 Hz, 0.1 m 1-σ error
USBL Transponder Communication Range	6000 m
USBL Localisation Accuracy in 1000 m	2.7 m 1-σ error
Number of AUVs positioned by the USBL in a single TDMA frame	10 AUVs
USBL TDMA Frame length	1 s
USBL update rate	4 s
Number of NBs	10 AUVs
NBs broadcasting period	1 s

**Table 3:** Intra-swarm communication modem and channel parameters.

Parameter	Value
Communication modem Frequency band	160 kHz
Communication data rate	50 kbit/s
Navigation aid length and duration	20 bytes; 3.2 ms
Navigation aid allocated TDMA time-slot length	20 ms
Noise level	60 dB
Water salinity	35 ppt
Water temperature	10 ${}^{\circ }$C
Rician fading parameter	10
Fast fading	enabled

## Appendix A

### Appendix A.1.

1. IF D is Shallow AND R is Short THEN Y is $L_{1}$.
2. IF U is Not Available AND G is Not Enough THEN Y is $L_{1}$.
3. IF B is Low AND U is Available AND G is Not Enough AND R is Long THEN Y is $L_{2}$.
4. IF B is Low AND U is Not Available AND G is Enough AND R is Long THEN Y is $L_{3}$.
5. IF U is Available AND R is Long THEN Y is $L_{2}$.
6. IF B is High AND U is Available AND G is Not Enough AND R is Mid THEN Y is $L_{2}$.
7. IF B is High AND U is Not Available AND G is Enough AND R is Long THEN Y is $L_{3}$.
8. IF D is Shallow AND B is High AND U is Not Available AND G is Enough AND R is Mid THEN Y is $L_{3}$.
9. IF D is Shallow AND B is High AND U is Available AND G is Enough AND R is Mid THEN Y is $L_{2}$.
10. IF U is Available AND R is Mid THEN Y is $L_{2}$.
11. IF B is Low AND U is Available AND G is Enough AND R is Long THEN Y is $L_{3}$.
12. IF D is Deep AND B is High AND U is Available AND R is Mid THEN Y is $L_{2}$.
13. IF D is Deep AND B is High AND U is Available AND R is Long THEN Y is $L_{2}$.
14. IF D is Shallow AND B is High AND U is Available AND R is Long THEN Y is $L_{2}$.
15. IF B is High AND U is Not Available AND G is Enough AND R is Long THEN Y is $L_{3}$.
16. IF D is Deep AND B is High AND U is Not Available AND G is Enough AND R is Mid THEN Y is $L_{1}$.
17. IF B is High AND U is Available AND R is Long THEN Y is $L_{2}$.
18. IF B is High AND U is Available AND R is Mid THEN Y is $L_{2}$.
19. IF B is High AND U is Not Available AND G is Enough AND R is Long THEN Y is $L_{3}$.
20. IF U is Not Available AND G is Enough AND R is Mid THEN Y is $L_{3}$.
21. IF D is Deep AND U is Available THEN Y is $L_{2}$.
22. IF D is Deep AND U is Available THEN Y is $L_{2}$.
23. IF D is Deep AND U is Not Available AND G is Enough AND R is Short THEN Y is $L_{1}$.

### Appendix A.2.

The null hypothesis (HA1) and the alternate hypothesis (HA2) of the one-tail two sample *t*-test performed in Section 4.4 are listed as follows:
**Hypothesis** **A1 (HA1).** Mean_EKF_Error = Mean_Fuzzy_Error.
**Hypothesis** **A2 (HA2).** Mean_EKF_Error > Mean_Fuzzy_Error.

**Table A1:** One-tail two sample *t*-test.

Swarm Size	H0 REJECTED	*p*-Value	Degree of Freedom	*t*-Statistics	Critical Value
50	No	1.0000	75.45	−4.35	1.6653
100	Yes	0.0252	194.48	1.96	1.6527
150	Yes	0.0324	255.45	1.85	1.6508

where the critical value is the inverse cumulative density function (CDF) of *t* distribution at 0.05 significant level. Rejection of the null hypothesis HA1 indicates that the mean localisation error of EKF-based method is greater than that of the proposed fuzzy-based method at 0.05 significance level.
